# An unusual delayed rectal metastasis from prostate cancer masquerading as primary rectal cancer

**DOI:** 10.1016/j.ijscr.2022.107732

**Published:** 2022-10-11

**Authors:** Mutahar Ali Tunio, Almootazbellah M. Agamy, Neil Fenn, Daniel Hanratty, Namor Wyn Williams

**Affiliations:** a Clinical Oncology, Swansea Bay University Health Board, Swansea, UK; b Internal Medicine, Swansea Bay University Health Board, Swansea, UK; c Urology, Swansea Bay University Health Board, Swansea, UK; d Colorectal Surgery, Swansea Bay University Health Board, Swansea, UK; e Histopathology, Swansea Bay University Health Board, Swansea, UK

**Keywords:** Prostate cancer, Rectal metastasis, Rare

## Abstract

**Introduction and importance:**

Rectal metastasis of prostate cancer origin is exceedingly rare. Its clinical presentation, endoscopic morphology, and histopathology are similar to primary rectal cancer. Misdiagnosis may lead to inappropriate treatment.

**Case presentation:**

We report a case of a gentleman in his 80's with a history of treated prostate cancer T3aN0M0 with radical prostatectomy sixteen years ago. He presented with one-year complaints of altered bowel habits and weight loss. Physical and rectal examination was unremarkable. Colonoscopy manifested some inflammatory changes in the rectum. The pelvis magnetic resonance imaging (MRI) showed an abnormal posterior rectal wall thickening 2 cm above the anal canal. Biopsy confirmed poorly differentiated adenocarcinoma of prostate origin. The staging workup was negative for other distant metastasis. After a multidisciplinary decision, the patient was started on androgen deprivation therapy and given palliative radiotherapy to the rectum. Six weeks later, the patient was stable with mild radiation proctitis.

**Clinical discussion:**

Management of rectal metastasis varies depending on the patient's choice, the extent of metastatic burden, symptoms, age, life expectancy, quality of life and comorbidities. While surgery remains the standard of care, other option including radiotherapy, hormonal therapy and chemotherapy has been documented in the literature with survival of few weeks to 2 years.

**Conclusion:**

Delayed rectal metastasis of prostate cancer after radical prostatectomy is a rare entity. Its clinical presentation and endoscopic and histopathological findings of rectal metastasis are similar to primary colorectal cancer, making diagnosis more demanding.

## Highlights

• Rectum is a rare isolated site of metastasis from prostate cancer.
• Symptoms and endoscopic morphology of rectal metastasis are similar to primary rectal cancer.
• Prostate-specific antigen is not always raised in rectal metastasis.
• Emergent lower gastrointestinal symptoms in patients with a history of prostate cancer warrants a multidisciplinary approach to reach an accurate diagnosis and prompt treatment.
• Management of rectal metastasis depends on patient desire, the severity of symptoms, age, quality of life, life expectancy and comorbidities.

## 1 Introduction

Prostate cancer is the second most predominant malignancy diagnosed in men globally, after lung cancer, with 52,300 cases every year (2016–2018) [1]. Metastatic disease is the leading cause of prostate cancer-associated deaths accounting for 13 % of all cancer deaths in men in the UK (2018) [2]. Regional lymph nodes are often the first site of metastasis, followed by metastases to the liver, lungs, and bones; however, the metastatic involvement of the rectum deriving from prostate cancer is a rare manifestation [3]. Due to anatomical adjacency, the rectum is predisposed to locoregional penetration through Denonvilliers' fascia by locally advanced prostate cancer in 1–12 % of cases [3]. Other postulated routes are lymphatic spread through the pelvic lymph node basins; and implantation along a trans-rectal needle biopsy tract in the rectum [4]. Prevailing lower gastrointestinal (GI) symptoms, the demonstration of a rectal mass on colonoscopy in the absence of prostate-specific antigen (PSA) rise in many cases, rectal metastases are mis-diagnosed as primary rectal cancers resulting in erroneous management [5]. Lane Z et al. who studied 23 patients with colorectal metastasis, reported that 19 of them had a prostate cancer diagnosis 2–18 years before colorectal metastasis, in contrast the remaining four patients had initially presented with lower GI symptoms [6].

We present a case of biopsy-proven rectal metastasis of prostate cancer origin in an elderly gentleman who underwent radical prostatectomy sixteen years ago for localized prostate cancer. This case has been reported in line with the SCARE 2020 criteria and PROCESS guidelines [7], [8].

## 2 Case presentation

A gentleman in his 80's underwent radical prostatectomy and lymphadenectomy in 2005 due to prostate adenocarcinoma pT3aN0M0. In 2007, his prostate-specific antigen (PSA) relapsed and was treated with salvage prostate radiotherapy and androgen deprivation therapy (ADT). Since then, he became incontinent and underwent implantation of an artificial sphincter in 2009. Sixteen years after radical prostatectomy, in September 2021, he presented with one-year history of altered bowel habits and rectal bleeding. He had also lost a notable amount of weight, approximately two stones over a year. On physical examination, Eastern Cooperative Oncology Group (ECOG) performance status was 1–2; his abdomen was soft and non-tender with no palpable masses or visceromegaly. A digital rectal examination (DRE) revealed a smooth feeling rectal mucosa but no perceptible craggy pelvic mass.

## 3 Investigations

**Fig. 1:**
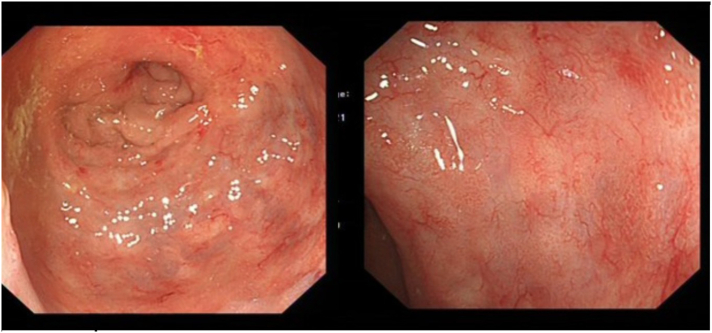
Colonoscopy showing inflammatory changes within the rectum, but showed no obvious mass.

**Fig. 2:**
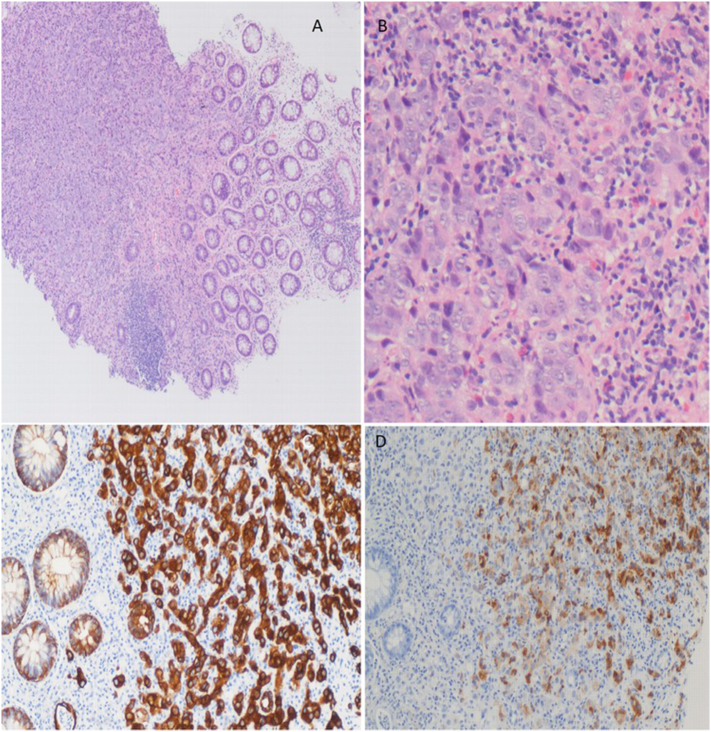
Biopsies from the inflamed area shows (A) ×40 of rectal mucosa and submucosa with adenocarcinoma infiltration, (B) ×100 with tumor cells showing prominent nucleoli and normal large bowel crypt on bottom left, (C) ×100 immunohistochemical CK positivity and (D) positive PSA immunohistochemical staining were suggesting prostate poorly differentiated adenocarcinoma of prostate origin.

**Fig. 3:**
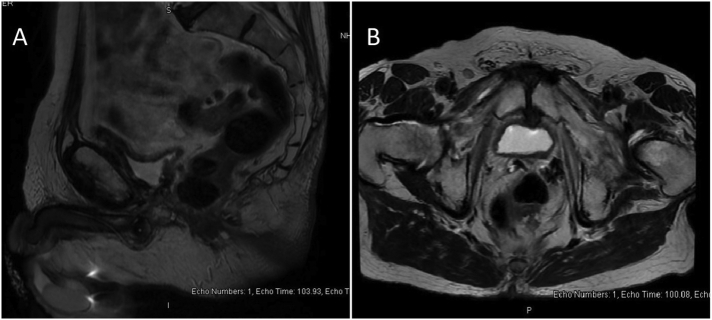
Pelvic magnetic resonance imaging (A) sagittal, (B) axial T2-weightted views showing abnormal posterior wall thickening within the lower rectum, around 2 cm above the anal canal and irregular outlines within the mesorectal soft tissue.

PSA was 4.7 microgram per litre (μg/L), and carcinoembryonic antigen (CEA) was normal (2 μg/L). Patient underwent a Faecal Immunochemical Test (FIT) test which was >400 microgram of Haemoglobin per gram of faeces (normal <10 μg Hb/g), and went on to have a screening colonoscopy, which showed some inflammatory changes within his rectum, which were thought to be secondary to radiation proctitis but showed no other colorectal pathology (Fig. 1). Biopsies taken from the inflamed region within his rectum showed poorly differentiated adenocarcinoma. Immunohistochemical stains of CK7 and PSA were positive suggestive of prostate adenocarcinoma as the primary origin (Fig. 2). Pelvic magnetic resonance imaging (MRI) showed abnormal posterior wall thickening within the lower rectum, around 2 cm above the anal canal; there were irregular outlines with some adjacent soft tissue (Fig. 3). Staging computed tomography (CT) of thorax, abdomen and pelvis was negative for any distant metastasis.

## 4 Treatment

After a multidisciplinary meeting, ADT was commenced and had prescribed palliative radiotherapy 20 Gy in five fractions over a week due to his age for the symptom control.

## 5 Outcome and follow-up

At the time of submission, patient was reviewed in the clinic six weeks after the completion of radiotherapy, complained of grade 2 radiation proctitis for which symptomatic medications were given and planned for follow-up at 3 months.

## 6 Discussion

Winter CC et al. first described three cases of non-contiguous involvement of the rectosigmoid region [9]. Since then, few cases have published rectal metastasis from prostate cancer, as shown in Table 1 [4], [5], [9], [10], [11], [12], [13], [14], [15], [16], [17], [18], [19], [20].

**Table 1:** Previously published cases reporting rectal metastasis from the prostate cancer.

Case report	Number	Age (years)	Treatment of primary	Presentation of rectal metastasis	Synch/Metach	Interval between primary and rectal metastasis	Colonoscopy findings	Other sites of metastasis	Treatment of rectal metastasis	Overall survival
Vaghefi H, et al [4]	1	69	Radical prostatectomy	Chronic constipation	Metach	5 years	Submucosal mass	None	Local excision	Several months
Wadehra A, et al [5]	1	90	Active surveillance	chronic constipation and poor appetite, rectal pain	Metach	NM	ulcerated, friable rectal mass 8 cm from the anal verge	None	Palliative care	NM
Winter CC, et al. [9]	3	Median 65	BSO, oral oestrogen	Weight loss	Synch	–	NM	Bones	BSO, oral oestrogen	12 months
Hamada Y, et al [10]	1	73	ADT	asymptomatic	Metach	NM	A flat elevated lesion in, 15 mm in size, in the rectum	NM	chemotherapy	NM
Venara A, et al [11]	1	75	Radical prostatectomy, bilateral ilio-obturator LND + salvage RT	Abdominal ache	Metach	20 years	NM	NM	AR	12 months Recurred at stump treated with RFA
Dulskas A, et al [12]	1	67	Retropubic prostatectomy and ilio-obturator LND + salvage RT	Dyschezia, pain in anal canal, and rectal bleeding	Metach	2 years	Anal fissure with ulceration on inspection	None	APR	NM
Nwankwo N, et al [13]	1	69	RT, bilateral orchiectomy, flutamide, salvage prostate cryoablation	Abdominal aches, rectal bleeding	Metach	12 years	3 cm oozing soft mass	NM	NM	NM
Morita T, et al [14]	1	61	ADT	Chronic constipation	Metach	5 months	Annular stricture	NM	ADT + chemotherapy and total pelvic exenteration and colostomy	NM
Fujita T, et al. [15]	1	77	ADT	Abdominal pain and obstructive symptoms	Synch	–	Small depressed lesions at the sigmoid colon and submucosal tumor at the rectum	NM	ADT	NM
Liu ZH, et al [16]	1	73	Bilateral orchiectomy, bicalutamide,	Altered bowel habits and rectal bleeding	Metach	6 years	2.5 × 3.5-cm elevated lesion of thickened mucosa with ill-defined margins	None	Pelvic CRT followed by AR	NM
Abbas TO, et al [17]	1	60	ADT	Weight loss, abdominal pain, Rectal bleeding and vomiting	Synch	–	Distal rectal sessile mass lying about 15 cm anal verge	Bones	ADT	NM
Culkin DJ, et al. [18]	6	Median 73	NM	Bowel obstruction	Synch (2) Metach (4)	NM		Bones (3) liver (3) None (2)	Colostomy (2) APR (1) AR (1) ADT (1) RFA (1)	1 week (1) 2 months (1) 6 months (2) 18 months (1) 25 months (1) (overall survival 9.3 months)
You JH, et al. [19]	1	78	ADT	Chronic constipation	Synch	–	Narrowing of the rectal lumen with normal overlying mucosa	NM	Colostomy, ADT	24 months
Dumontier I, et al [20]	1	84	ADT	NM	Metach	2 years	NM	NM	Colostomy, palliative care	3 months

Synch = synchronous; Metach = metachronous; NM = not mentioned; AR = anterior resection; APR = abdominoperineal resection; ADT = androgen deprivation therapy; CRT = chemoradiation; RFA = radiofrequency ablation; BSO = bilateral subcapsular orchiectomy.

The lower gastrointestinal tract is a rare site of metastasis. The incidence of metastasis to the lower gastrointestinal tract is 0.05 % of all metastases [11], [12]. Rectal Metastasis are particularly scarce, and its true incidence is unrevealed. The most common origins of rectal metastasis are breast, stomach, gynecologic cancers and prostate [11], [12], [13].

The median age of prostate cancer patients with rectal metastasis was 73 years (range: 60–90). The median time between primary and rectal metastasis was 6.7 years. Serum PSA was normal in eight (38.1 %) patients at presentation. Total thirteen (61.9 %) were metachronous, and eight were synchronous (38.1 %). Six patients (28.6 %) had other metastases in addition to rectal metastasis. The presentation of rectal metastasis was variable with frequency; obstructive symptoms (*n* = 7;33.3 %) chronic constipation (*n* = 4; 19.1 %), abdominal pain (n = 4; 19.1 %), rectal bleeding (n = 4; 19.1 %) and asymptomatic (*n* = 1; 4.8 %). As these symptoms are customarily associated with primary rectal cancer, patients may initially be mismanaged and have resultant hampers in care [7], [19], [20]. Further, rectal metastasis from prostate cancer had colonoscopic similarities with primary rectal cancer; rectal mass (*n* = 5; 23.8 %) 3–15 cm anal verge, ulceration (*n* = 2; 9.5 %) as in our case and stricture/significant narrowing of rectal lumen (*n* = 2; 9.5 %). Histopathologically, thirteen (61.9 %) had poorly differentiated adenocarcinoma with PSA immunohistochemical positivity in 8 patients (38.1 %).

Management of rectal metastasis was variable depending on the patient's choice, the extent of metastatic burden, symptoms, age, life expectancy, quality of life and comorbidities. Local treatment was mainly in the form of surgery; local excision (*n* = 1; 4.8 %), anterior resection (*n* = 3; 14.3 %), Abdominoperineal resection (n = 2; 9.5 %), pelvic exenteration (n = 1; 4.8 %) and colostomy (*n* = 4; 19.1 %). One (4.8 %) patient experienced anastomotic recurrence salvaged with radiofrequency ablation [18]. As in our case, one patient (4.8 %) received radiotherapy. Five (23.8 %) patients got androgen deprivation therapy; two (9.5 %) patients underwent systemic chemotherapy, and the best supportive care for two (9.5 %) patients. Overall survival was between two weeks to 25 months.

In conclusion, Rectal metastasis of prostate cancer origin is a rare entity. Its clinical presentation and endoscopic and histopathological findings of rectal metastasis are similar to primary colorectal cancer, making diagnosis more demanding. Thus patients with a history of prostate cancer with emergent rectal symptoms, a multidisciplinary approach is crucial to reach an accurate diagnosis and prompt treatment.

## Consent

Written informed consent was obtained from the patient for publication of this case report and accompanying images. A copy of the written consent is available for review by the Editor-in-Chief of this journal on request.

## Ethical approval

Nil.

## Funding

Nil.

## Author contribution

Neil Fenn (conceptualization, data curation, writing original draft and review), Mutahar A, Tunio (data collection, writing and formal analysis), Almootazbellah M. Agamy (data collection, investigation, analysis and draft review), Daniel Hanratty (data collection, methodology, writing, draft review/editing), Namor Wyn Williams (data collection, visualization, writing editing, draft review).

## Guarantor

Mutahar A. Tunio.

## Research registration number

Not applicable.

## Declaration of competing interest

None to declare.
